# ABO gene polymorphisms: a molecular bridge linking disease susceptibility to therapeutic outcomes

**DOI:** 10.3389/fmed.2026.1796826

**Published:** 2026-06-17

**Authors:** Shaoqing Yin, Jiaye Li, Wei Zhang, Rui Ding, Yan Li, Kai Wang, Wenjian Jiang, Yi-Da Tang, Zhengjun Yi, Yaohua Zhang, Meng Li

**Affiliations:** 1 School of Medical Laboratory, Shandong Second Medical University, Weifang, China; 2 Department of Cardiology and Institute of Vascular Medicine, State Key Laboratory of Vascular Homeostasis and Remodeling, Peking University Third Hospital, Beijing, China; 3 NHC Key Laboratory of Cardiovascular Molecular Biology and Regulatory Peptides, Peking University, Beijing, China; 4 Weifang Second People's Hospital, Weifang, China; 5 Department of Emergency Medicine, Peking University Third Hospital, Beijing, China; 6 Department of Physiology and Pathophysiology, School of Basic Medical Sciences, State Key Laboratory of Vascular Homeostasis and Remodeling, Beijing Advanced Center of Cellular Homeostasis and Aging-Related Diseases, Peking University, Beijing, China; 7 Department of Cardiac Surgery, Beijing Anzhen Hospital, Capital Medical University, Beijing, China; 8 Beijing Lab for Cardiovascular Precision Medicine, Beijing, China; 9 Research Unit of Medical Science Research Management/Basic and Clinical Research of Metabolic Cardiovascular Diseases, Chinese Academy of Medical Sciences, Beijing, China; 10 Beijing Key Laboratory of Clinical Evaluation of Cardiovascular-Kidney-Metabolic and Immuno-Inflammatory Innovative Drugs and Medical Devices, Beijing, China

**Keywords:** ABO blood group, biomarkers, disease susceptibility, drug response, glycosyltransferases, precision medicine

## Abstract

ABO blood group antigens represent more than surface markers on red blood cells. They are pivotal genetic determinants that affect susceptibility to various diseases and variations in drug response. Polymorphisms in the ABO gene produce glycosyltransferases with distinct structures and functions, leading to differential risk for cancers, cardiovascular diseases, and infectious diseases. The ABO gene exerts its profound influence on disease pathogenesis primarily through the regulation of ABH antigens, interfacing with critical pathophysiological pathways such as coagulation, inflammation, and cellular signaling. Epidemiological data link non-O blood groups with heightened incidence of gastric, pancreatic, ovarian, and bladder cancers. Individuals with non-O blood groups present higher concentrations of von Willebrand factor (vWF), which predisposes them to atherosclerosis, thrombosis, and ischemic heart disease. This contrasts sharply with the risk profile of group O individuals, who show a greater likelihood of contracting gastrointestinal infections—notably from Helicobacter pylori, noroviruses, and Vibrio cholerae—and tend to suffer from more severe symptoms. Furthermore, functional variations in ABO glycosyltransferases can directly modulate drug efficacy. Consequently, the development of predictive models for disease risk and treatment response based on ABO blood typing holds significant promise for advancing personalized prevention and therapeutic strategies for cancer, cardiovascular, and infectious diseases.

## 1 Introduction

Since Karl Landsteiner's discovery of the ABO blood group system in 1900, this classical genetic polymorphism has remained a cornerstone of transfusion medicine and immunological research (1, 2). Over the decades, new blood group systems have been continuously reported. As of June 2025, the International Society of Blood Transfusion (ISBT) recognizes 48 distinct human blood group systems (3). Major human blood group systems include ABO, Rh, Lewis, Kell, and others. This review primarily focuses on the ABO blood group system (4). The ABO blood group system, one of the most clinically critical human polymorphisms, is determined by glycosyltransferases encoded by the ABO gene (5). These enzymes catalyze the modification of the H antigen to produce the characteristic A, B, or O antigens. These glycans are not only confined to red blood cells but are also broadly expressed on vascular endothelial cells, platelets, and epithelial cells, and are also secreted into bodily fluids like saliva and sweat (6, 7). Functionally, ABO antigens are involved in pivotal physiological and pathological processes such as coagulation (8), immune regulation (9–11), pathogen adhesion (12, 13), and inflammatory responses (14–17). This extensive involvement underpins their profound impact on the pathogenesis and progression of diverse diseases.

With innovations in molecular biology and epidemiological methods, researchers' understanding of blood group-related diseases continues to deepen. Since its discovery over a century ago, the biological implications of the ABO system extend well beyond transfusion and transplantation medicine to include roles in a wide array of diseases (18). For instance, individuals with non-O blood types demonstrate increased vulnerability to a range of diseases spanning cancer to cardiovascular disorders, primarily due to their inherently higher levels of vWF (19). At the molecular level, histo-blood group ABO system transferase (BGAT), the product of the ABO locus, catalyzes the addition of A or B antigens to VWF, prolonging vWF half-life and elevating plasma levels of vWF and coagulation factor VIII (FVIII). In the HUNT study, elevated plasma BGAT levels were associated with an increased risk of venous thromboembolism (VTE), and this association remained significant after adjustment for vWF and FVIII, suggesting that BGAT-mediated glycosylation contributes to thrombosis risk through both vWF/FVIII-dependent and additional mechanisms (e.g., altered vWF proteolysis or modulation of other hemostatic and inflammatory proteins) (20). Through these pathways, this shared mechanism facilitates cancer progression via pathways such as heightened cell adhesion and inflammatory reactions, while simultaneously elevating thrombosis risk through accelerated clotting processes (21, 22). Moreover, blood group antigens—specifically the H antigen—function as direct pathogen receptors in infectious diseases. For instance, *H. pylori* can bind to the H antigen (23), and noroviruses utilize H antigen glycans for host infection (24), suggesting a potential role for ABO antigens in mediating pathogen–host interactions.

Beyond its established role in disease risks, the ABO blood group is increasingly recognized for its pronounced implications in clinical drug efficacy. Studies indicate that patients with blood type O require lower maintenance doses of warfarin to achieve target international normalized ratio (INR) levels compared to non-O individuals (25). The ABO blood group influences the variable response to the antiplatelet agent clopidogrel (26). Furthermore, significant differences in therapeutic efficacy and adverse reaction risks have also been documented across blood groups for anti-tuberculosis drugs and various anti-cancer therapies (27, 28). Consequently, incorporating ABO blood typing into clinical practice holds substantial promise for developing personalized therapeutic interventions and enhancing overall drug response rates.

**Figure 1:**
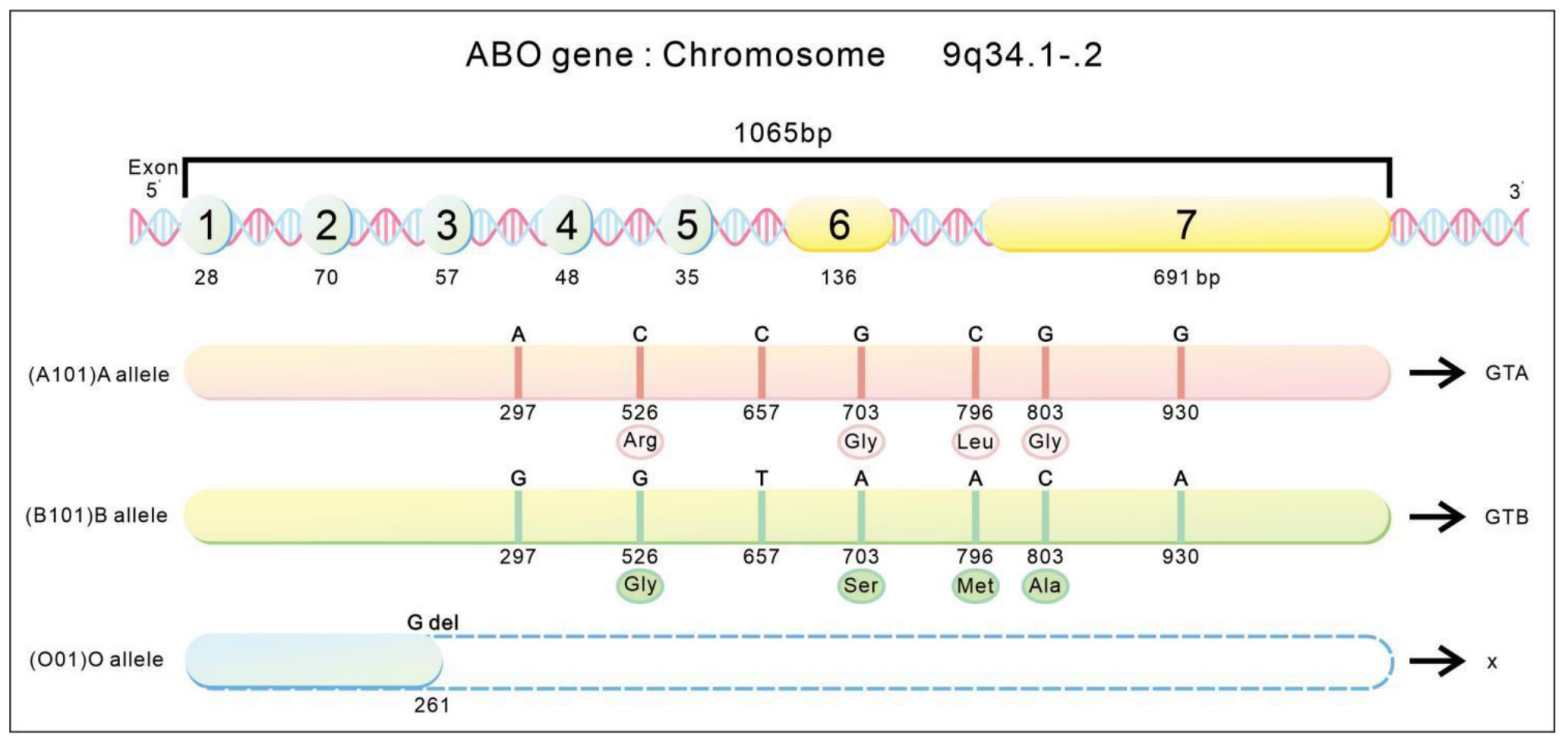
Genetic basis of the ABO blood group system. The ABO blood group system is defined by allelic variants of the ABO gene on chromosome 9, which encode glycosyltransferases responsible for A and B antigen synthesis. The A allele produces an α-1,3-N-acetylgalactosaminyltransferase (GTA), whereas the B allele carries critical nucleotide substitutions that confer α-1,3-galactosyltransferase (GTB) activity with distinct substrate specificity. The common O allele harbors a frameshift mutation (c.261delG), yielding a truncated and non-functional protein.

The rise of precision medicine has highlighted the importance of individual genetics in healthcare. Yet, the ABO blood group—a simple, universal genetic trait—remains underutilized in clinical practice, even as complex omics technologies advance. This review explores the genetic basis of ABO and compiles its wide-ranging links to disease risk, progression, and drug response. It argues for the integration of ABO typing into a cohesive clinical framework that unifies prediction and treatment, opening new paths for personalized risk assessment and therapy. A systematic literature search of the PubMed database was conducted, identifying 919 records. Additional records identified through other sources (*n* = 36). After removal of 10 duplicates, 945 records underwent title and abstract screening. A total of 665 records were excluded at this stage for the following reasons: not published in English (*n* = 68), animal or cellular studies (*n* = 48), case reports (*n* = 52), conference abstracts (*n* = 3), letters/editorials (*n* = 15), or topics unrelated to the ABO-disease interface (*n* = 479). Full texts were sought for the remaining 280 reports, of which 43 could not be retrieved. Following a detailed full-text eligibility assessment of 237 reports, 198 were further excluded owing to incompatible research design (*n* = 69) or insufficient ABO-stratified outcome data (*n* = 129). A final set of 39 studies formed the evidence base for this review. The PRISMA flow diagram illustrating this selection process is presented in Figure 1, with the complete search strategy detailed in Supplementary File 1.

## 2 Molecular basis of ABO blood groups

### 2.1 The ABO gene directly determines glycosyltransferase specificity

As the key genetic factors determining the ABO blood group system, ABO alleles encode blood group glycosyltransferases whose functional variations dictate an individual's blood group phenotype. The human ABO gene is located on the long arm of chromosome 9 (9q34.1-q34.2), spanning over 18 kb, and it comprises at least seven exons. Among them, exons 6 and 7 are particularly critical, which encode approximately 91% of the glycosyltransferase catalytic domain. This region constitutes the core functional segment that determines the enzyme's biological activity (29, 30). The genetic foundation of the ABO system is primarily established by three classical alleles: A101, B101, and O01. The canonical A allele (A101) encodes a fully functional α-1,3-N-acetylgalactosaminyltransferase (GTA). The canonical B allele (B101) differs from A101 by four crucial nucleotide substitutions (c.526C>G, c.703G>A, c.796C>A, c.803G>C), resulting in four amino acid changes (p.Arg176Gly, p.Gly235Ser, p.Leu266Met, p.Gly268Ala) (31). These mutations alter the enzyme's specificity, creating an α-1,3-galactosyltransferase (GTB). The primary O allele (O01) contains a single-base deletion (c.261delG), leading to a frameshift and the production of a truncated, enzymatically inactive protein that lacks GTA or GTB function (32, 33). At its core, the ABO system is defined by the action of its specific glycosyltransferases, which are ultimately responsible for producing the ABH antigens (Figure 1).

### 2.2 ABO glycosyltransferases determine ABO blood group antigens

**Figure 2:**
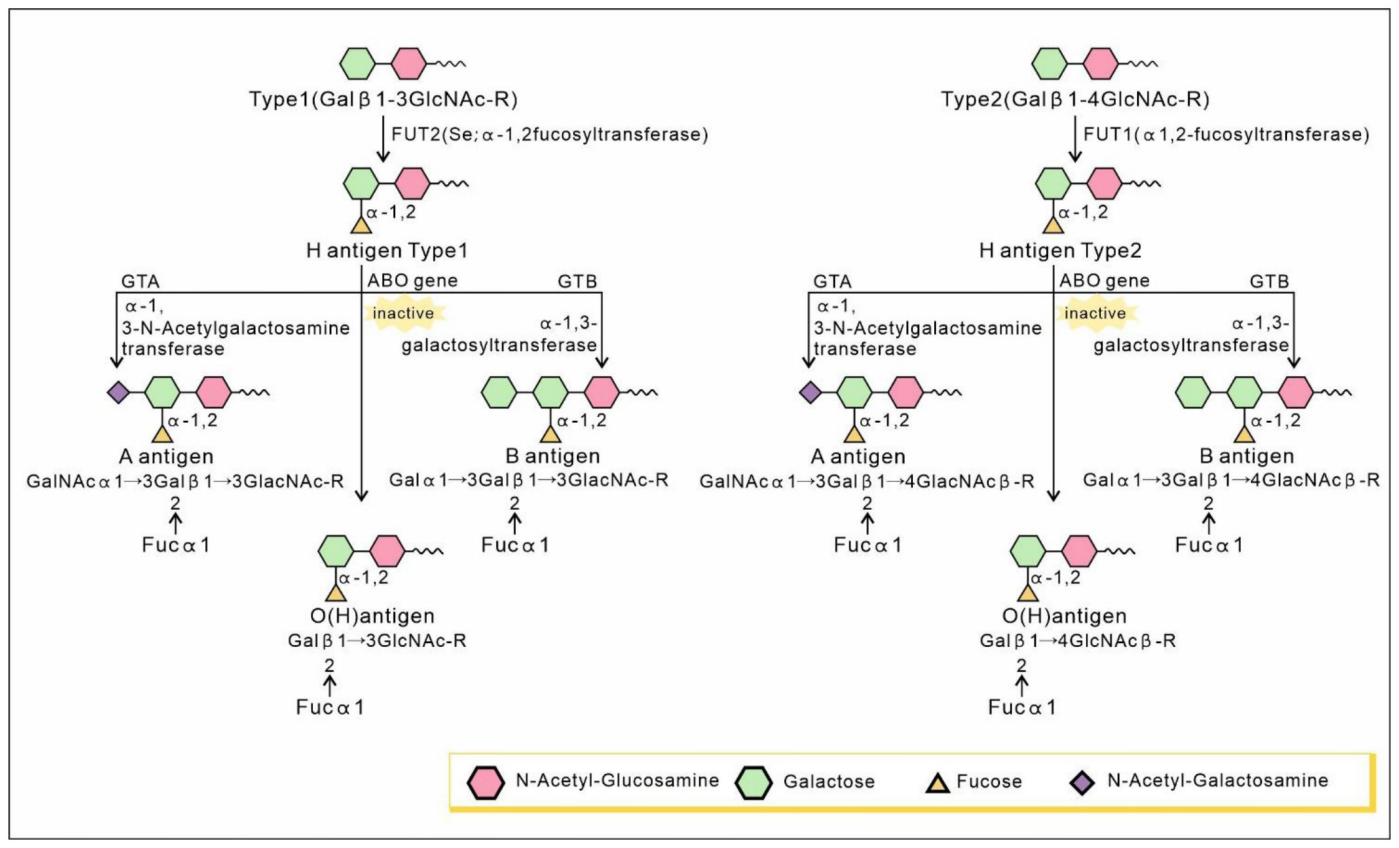
Biosynthesis and classification of ABH antigens. The ABH antigens are synthesized from the H antigen precursor, which exists in membrane-bound and secreted forms. The membrane-bound form (type II H) is synthesized by FUT1 on type 2 chains, while the secreted form (type I H) is produced by FUT2 on type 1 chains. The A or B allele-encoded glycosyltransferases (GTA or GTB) then modify the type-specific H antigen to produce the corresponding A or B antigen.

The ABH antigens constitute the structural core of the ABO blood system and are classified as either membrane-bound or secreted based on their cellular localization. A fundamental aspect of their biology is that both classes are derived from the H antigen, which serves as the indispensable biosynthetic precursor (34). The synthesis of the H antigen precursor is compartmentalized by two distinct fucosyltransferases: FUT2 acts on type 1 chains (Galβ1 → 3GlcNAc-R) to create the secreted H antigen (type I H antigen), while FUT1 modifies type 2 chains (Galβ1 → 4GlcNAc-R) to form the membrane-bound version (type II H antigen), effectively defining the antigen's localization (35). The final determinant of ABO blood type lies in the subsequent modification of these H antigens by the specific glycosyltransferases encoded by the ABO gene. The GTA enzyme, encoded by the A allele, transfers α-N-acetylgalactosamine to the terminal galactose of the H antigen, forming the A antigen. Similarly, the GTB enzyme, encoded by the *B* allele, transfers α-D-galactose to the same position, generating the B antigen (36–38). Consequently, secreted A and B antigens are synthesized from type I H antigen, whereas their membrane-bound counterparts originate from type II H antigen (Figure 2) (39). While membrane-bound antigens primarily determine red blood cell phenotypes and mediate immune recognition, their secreted counterparts, present in bodily fluids, are key players in microbial defense and in regulating host susceptibility to a spectrum of conditions (40–42).

The diversity of the ABO system, which extends beyond the common A, B, AB, and O types, is driven by extensive genetic polymorphism. This gives rise to numerous subtypes, such as A1, A2, Ax, and Aend for group A, and B3, Bx, Bm, Bel, and B(A) for group B (43, 44). The formation of these ABO subtypes originates from the genetic polymorphism of the ABO gene. Essentially, genetic mutations—affecting both the coding regions and regulatory sequences like the promoter—lead to structural and functional abnormalities in the ABO glycosyltransferases.

**Figure 3:**
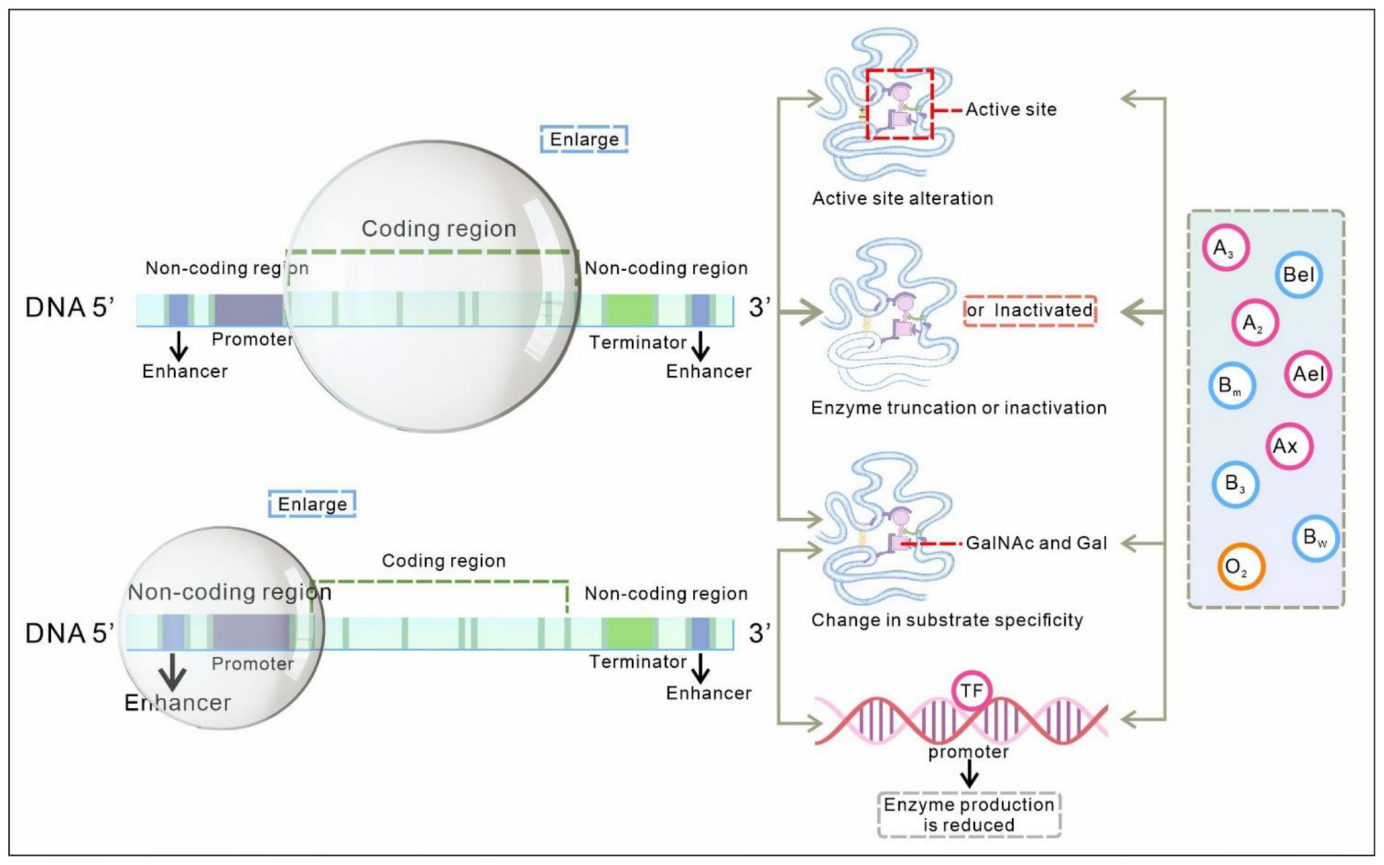
Genetic mechanisms underlying ABO subtypes. ABO subtypes arise from genetic polymorphisms that compromise enzyme function or expression. Coding region mutations can disrupt the active site or introduce premature stop codons, while regulatory mutations can reduce transcription. These alterations lead to reduced enzymatic activity, abnormal kinetics, or altered substrate specificity, resulting in weakly expressed or atypical antigen.

The dysfunction of ABO glycosyltransferases due to coding region mutations occurs via two main pathways. Mechanistically, some mutations disrupt critical domains within the enzyme's active site, impairing substrate binding or catalysis (45–47). Others introduce a premature termination codon (PTC), resulting in a truncated protein or triggering nonsense-mediated mRNA decay, which abolishes protein production (46, 48–50). Conversely, non-coding region mutations typically diminish gene expression by hindering transcription factor binding (9, 51–53). Additionally, mutations in either region can perturb the enzyme's kinetic parameters (e.g., Km, Vmax), thereby modifying substrate specificity. Consequently, these alterations manifest phenotypically as either weak subgroups with reduced antigen density or as atypical antigen expressions (54). Together, these diverse mutational effects intricately shape the complex genetic and phenotypic landscape of the ABO system (Figure 3).

## 3 ABO blood group and disease susceptibility/progression

### 3.1 Cancer

#### 3.1.1 Gastric cancer

Substantial evidence confirms a pronounced association between the ABO blood group and gastric cancer risk (55–57). Individual risk differences are determined by key elements of the ABO genetic background-including specific genotypes, Single Nucleotide Polymorphisms (SNPs), and allele dosage effects-acting in combination. A prospective study by Nakao et al. revealed a clear link between ABO genotype and gastric cancer risk. Compared with individuals with blood type B (BO/BB genotype) or blood type O, those with blood type A (AA genotype) exhibited a remarkable higher risk of gastric cancer (BO vs. AA: OR = 0.53, 95% CI: 0.36–0.77; BB vs. AA: OR = 0.48, 95% CI: 0.23–1.02; OO vs. AA: OR = 0.70, 95% CI: 0.50–0.99). The study further confirmed a dosage effect of the A allele. Relative to individuals carrying non-A alleles (OO, BO, BB genotypes), the gastric cancer risk progressively increased for those carrying one and two A alleles (OR = 1.37, 95% CI: 1.13–1.67; OR = 1.59, 95% CI: 1.15–2.21) (58). A large-scale study and meta-analysis by Wang Z et al. corroborated these findings. Their case-control study (1,045 gastric cancer patients vs. 53,026 healthy controls) demonstrated an appreciable higher risk for individuals with blood type A (OR = 1.34, 95% CI = 1.25–1.44) and a considerably lower risk for blood type O individuals (OR = 0.80, 95% CI = 0.72–0.88). A subsequent meta-analysis (24 studies, 15,843 cases vs. 1,421,740 controls) validated this association in a broader population (Type A: OR = 1.11, 95% CI = 1.07–1.15; Type O: OR = 0.91, 95% CI = 0.89–0.94) (59).

Genomic studies have elucidated the association between the ABO locus and gastric cancer at a molecular level. Tanikawa et al. identified that SNP rs7849280, located in the 3′ flanking region of the ABO gene on 9q34.2, is closely associated with gastric cancer risk (*p* = 2.64 × 10^−13^, OR = 1.148), with its G allele constituting a risk allele (60, 61). This risk G allele exhibits strong linkage disequilibrium (LD) with the ABO A allele. The frequency of the G allele is 75.3% in individuals with blood type A, compared to only 0.6%-37.6% in those with O, B, or AB blood types. This genetic evidence supports the involvement of the ABO region in the elevated risk observed in individuals with blood type A, with rs7849280 serving as a tag SNP for the A allele (60, 61). The mechanistic basis of this association, however, requires direct investigation of ABO antigens rather than this polymorphism.

The ABO blood group is considered a genetic determinant in gastric cancer susceptibility. Blood type A and a higher dosage of the A allele have been associated with elevated disease risk, whereas blood type O has been linked to a protective effect.

#### 3.1.2 Pancreatic cancer

Pancreatic cancer is a highly malignant neoplasm with increasing global incidence and mortality (62). Recent studies have demonstrated a strong association between the ABO blood group system and the development, progression, and prognosis of this disease. In an analysis of 12 prospective cohorts including 1,534 pancreatic cancer cases and 1,583 healthy controls, Wolpin et al. (63) reported that, compared with individuals with blood type O, those with blood types A (OR = 1.38), AB (OR = 1.47), and B (OR = 1.53) conferred a higher risk of pancreatic cancer. Moreover, the risk increased in a dose-dependent manner with the number of non-O alleles carried (AO vs. OO: OR = 1.33; AA vs. OO: OR = 1.61; BO vs. OO: OR = 1.45; BB vs. OO: OR = 2.42). Further analysis of ABO allele subtypes showed that the A1 allele notably increased pancreatic cancer risk (A1/O genotype: OR = 1.48; A1/A1 genotype: OR = 1.71), whereas the A2 allele had no such effect (A2/O genotype: OR = 0.96). In contrast, the O01 and O02 alleles demonstrated comparable protective effects (64). These results suggest that pancreatic cancer susceptibility is determined not merely by ABO phenotype but by the distinct enzymatic activities of glycosyltransferases encoded by specific ABO allele subtypes.

Furthermore, the ABO blood group serves as an important prognostic indicator in pancreatic cancer. A study of 406 Japanese pancreatic cancer patients found that the median survival time was considerably shorter in carriers of the A allele than in non-carriers (361 days vs. 494 days, *p* = 0.048). This survival difference was more pronounced in the subgroup of patients who underwent surgical resection (A allele: 773 days vs. non-A allele: not reached, *p* = 0.031). Multivariate analysis confirmed the A allele as an independent risk factor for long-term prognosis (all *p* < 0.05) (65). To further clarify the relationship between ABO blood group and pancreatic cancer outcomes, Correa et al. conducted a systematic review and meta-analysis (18 studies, 9,084 patients). This analysis comprehensively evaluated key endpoints, including overall survival (OS), median survival time, tumor stage, lymph node metastasis, and distant metastasis. The results demonstrated a significant survival advantage in blood type O patients compared to non-O patients, conferring an unadjusted HR = 0.82 (95% CI = 0.75–0.91) and an adjusted HR = 0.76 (95% CI = 0.68–0.85). Additionally, type O patients were less likely to be diagnosed with advanced-stage (III-IV) disease (OR = 0.82, 95% CI = 0.69–0.97) (66).

At the level of SNPs and genetic variation, a genome-wide association study (GWAS) established that the rs505922 locus in the chromosomal 9q34 region, located within the first intron of the ABO gene, shows a marked association with pancreatic cancer risk (combined *p* = 5.37 × 10^−8^, OR = 1.20, 95% CI = 1.12–1.28). The T allele at this locus is in complete linkage disequilibrium (r^2^ = 1.0) with the O blood group allele, thereby offering compelling genetic evidence for the protective role of blood type O against pancreatic cancer (67). This finding has been corroborated in Asian populations. A case-control study in a Japanese cohort confirmed that individuals with blood type O exhibited a notably reduced risk of pancreatic cancer compared with those with non-O blood types. Specifically, the AO genotype (OR = 1.67, 95% CI = 1.08–2.57) and BB genotype (OR = 3.28, 95% CI = 1.38–7.80) were associated with significantly elevated risk. Furthermore, rs505922 again demonstrated strong linkage with the O allele (r^2^ = 0.96) in this population (68). Based on the above information, rs505922 is in strong linkage disequilibrium with the O blood group allele, so its association with pancreatic cancer risk likely reflects an indirect effect of blood group O itself.

In pancreatic carcinogenesis and prognosis, non-O blood types (especially the B blood group and the A1 allele) have been associated with an elevated risk, with a possible dose-dependent effect of the A1 allele. Furthermore, the presence of the A allele has been suggested as an indicator of less favorable prognosis, whereas blood type O has been linked to better survival outcomes. Epidemiological studies across diverse populations, together with corroborating genetic evidence, appear consistent with these associations.

#### 3.1.3 Ovarian cancer

Multiple studies have reported an association between ovarian cancer and a spectrum of ABO-related factors, including blood group, genotype, and specific genetic variants. Regarding disease susceptibility, an analysis by the Ovarian Cancer Association Consortium (OCAC) involving 5,233 cases and 6,838 controls indicated that blood type A was associated with a modest increase in ovarian cancer risk compared to type O (OR = 1.09, 95% CI = 1.01–1.18, *p* = 0.03) (69). A study by Wang C et al. focusing on epithelial ovarian cancer (EOC) in Chinese women further substantiated these observations. Blood type A was linked to a notably higher EOC risk (OR = 1.18, 95% CI = 1.03–1.36, *p* = 0.019). Furthermore, the rate of abnormal ABH antigen expression in tumor tissues was pronouncedly elevated in type A patients (76.5%) compared to type O (21.1%) and type B (5.0%) individuals (70). However, an inverse pattern emerged in relation to survival prognosis. A retrospective cohort study of 713 ovarian cancer patients demonstrated that type A patients experienced substantially prolonged OS relative to type O patients (HR = 0.75, 95% CI = 0.60–0.93) and all non-A patients (HR = 0.77, 95% CI = 0.63–0.94). This survival benefit was more evident in Caucasians. At the allele subtype level, carriers of the A2 allele exhibited the most favorable OS (HR = 0.50, 95% CI = 0.25–0.99) (71).

At the level of SNPs and genetic variation, a GWAS identified the rs633862 locus as a susceptibility locus for EOC among Han Chinese women. The minor A allele at this locus conferred a protective effect, associated with a meaningful reduction in EOC risk (OR = 0.83). This locus exhibits moderate linkage disequilibrium (LD = 0.57) with the ABO O allele. As a result, the protective effect conferred by the A allele is consistent with, and provides a genetic basis for, the reduced EOC risk clinically observed in individuals with blood type O (72). Further analysis revealed that the rs633862 polymorphism within the 9q34.2 region is also linked to ovarian cancer survival. Patients carrying the AG/GG genotypes demonstrated a clear survival advantage, exhibiting longer OS compared to those with the AA genotype (univariate HR = 0.69, 95% CI = 0.49–0.98, *p* = 0.035). Moreover, the rs633862 AA genotype correlated with increased ABO mRNA expression (*p* = 1.8 × 10^−13^), suggesting that this genetic variant may influence clinical outcomes by modulating ABO gene transcription (73).

These findings suggest a dual role for ABO blood group in ovarian cancer. While blood type A (notably the A1 allele) has been associated with increased disease susceptibility, the same blood group (especially the A2 allele) appears to be linked to a more favorable overall survival prognosis.

#### 3.1.4 Bladder cancer

Emerging evidence suggests a role for the ABO blood group system in bladder cancer development and outcomes, yet a consistent prognostic role has proven elusive. Illustrating this, a retrospective cohort study by Klatte T et al. involving 931 primary NMIBC (non-muscle-invasive bladder cancer) patients found blood type O was linked to elevated risks of recurrence and progression. Compared to this group, patients with blood type A exhibited a significantly lower risk of recurrence (SHR = 0.77, *p* = 0.015) and progression (SHR = 0.57, *p* = 0.031). A similar protective trend was observed for type B against recurrence (SHR = 0.60, *p* = 0.004) and progression (SHR = 0.42, *p* = 0.075). The predictive accuracy for recurrence and progression of bladder cancer was augmented by the incorporation of ABO blood group into multivariable models, with one measure for 12-month progression prediction increasing from 65.8% to 73.0% (74).

The protective association for non-O blood types was not universal. Contradictory evidence emerged from a study by Gershman B et al., which was limited to patients of Muscle-Invasive UC urothelial carcinoma (MIUC). In their cohort of 2,086 patients undergoing radical cystectomy for bladder urothelial carcinoma, non-O blood types were associated with adversely affected 5-year recurrence-free survival (RFS; 65% vs. 69%, *p* = 0.04) and cancer-specific survival (CSS; 64% vs. 70%, *p* = 0.02). Blood type A was independently correlated with heightened cancer-specific mortality (HR = 1.22, *p* = 0.01). Furthermore, in the subgroup with organ-confined disease (pT2N0), patients possessing non-O blood types exhibited significantly reduced 5-year RFS (75% vs. 82%, *p* = 0.002) and CSS (77% vs. 85%, *p* = 0.001) relative to type O patients (75).

Investigation at the genotype level has elucidated distinct associations between ABO alleles and clinical characteristics. One case-control study demonstrated that carriers of the O1, A1, and B alleles had a significantly reduced frequency of malignant tumors (*p* < 0.001, *p* < 0.001, *p* = 0.013, respectively). Paradoxically, the O1 and A1 alleles were simultaneously identified as adverse prognostic indicators (71.4%, 70.1%), being significantly associated with high-grade histology and elevated recurrence rates (70.2%, 74.2%) (*p* < 0.001, *p* = 0.019 and *p* < 0.001, *p* = 0.007, respectively). The A2 allele, however, presented a protective profile, marked by a significant absence of metastatic disease (0%, *p* = 0.024) (76). The connection between ABO blood group and bladder cancer prognosis is evident, yet the literature is marked by contradictory conclusions regarding the risk conferred by specific blood types. These variations in prognostic associations are likely attributable to differences in cohort demographics, disease stage at study, treatment modalities received, and analytical methodologies employed (Table 1).

**Table 1:** Summary of studies evaluating the relationship between ABO blood group and cancer.

Disease category	Study population	Associated blood type(s)/gene(s)	Aspect involved	Level of association^*^	Effect estimate (95% Cl)
Gastric cancer (GC)	703 GC patients vs. 1,465 controls (Japan) (58)	AA genotype	Susceptible	↑	BO vs. AA: OR = 0.53, 95% CI: 0.36–0.77; BO vs. AA: OR = 0.48, 95% CI: 0.23–1.02; OO vs. AA: OR = 0.70, 95% CI: 0.50–0.99 (*p* < 0.001)
	1,045 GC patients vs. 53,026 controls (China) (59)	Blood type A	Susceptible	↑	A vs. Non-A: OR = 1.34, 95% CI = 1.25–1.44
	15,843 GC cases vs. 1,421,740 controls (Meta-analysis) (59)	Blood type A	Susceptible	↑	A vs. Non-A: OR = 1.11, 95% CI = 1.07–1.15
Pancreatic cancer	1,534 PC patients vs. 1,583 controls (Multi-center) (63)	Non-O blood type	Susceptible	↑	A vs. O: OR = 1.38, 95% CI: 1.18–1.62 AB vs. O: OR = 1.47, 95% CI: 1.07–2.02 B vs. O: OR = 1.53, 95% CI: 1.21–1.92 O vs. Non-O: OR = 1.42, 95% CI: 1.22–1.65
		Non-O genotype	Susceptible	↑	AO vs. OO: OR = 1.33, 95% CI:1.13–1.58; AA vs. OO: OR = 1.61, 95% CI:1.22–2.18; BO vs. OO: OR = 1.45, 95% CI:1.14–1.85; BB vs. OO: OR = 2.42, 95% CI:1.28–4.57
	1,534 PC patients vs. 1,583 controls (Multi-center) (64)	A1 allele	Susceptible	↑	A1/O vs. OO: OR = 1.48,95%CI: 1.23–1.78; A1/A1 vs. OO: OR = 1.71,95% CI: 1.18–2.47
	406 PC patients (Japan) (65)	A allele	Prognosis/Median survival time (MST)	↓	A allele vs. the non-A allele: 361 days vs. 494 days, HR = 1.454,95%CI: 1.101–1.928
	9,084 PC patients (Global/Meta-analysis) (66)	blood type O	Prognosis/Overall survival (OS)	↑	O vs. Non-O: HR = 0.76,95% CI 0.68–0.85
			Prognosis/Tumor stage (stage III-IV)	↓	O vs. Non-O: OR = 0.82, 95% CI 0.69–0.97
Ovarian cancer	5,233 EOC cases vs. 6,837 controls (European ancestry) (69)	Blood type A/A genotype/A1 allele	Susceptible	↑	A vs. O: OR = 1.09, 95% CI = 1.01–1.18, *p* = 0.03; AO vs. OO: OR = 1.11, 95% CI = 1.01–1.22, *p* = 0.03; A1 allele vs. O allele: OR = 1.15, 95% CI = 1.02–1.30, *p* = 0.03
	1,870 EOC cases vs. 4,829 controls (China) (70)	Blood type A	Susceptible	↑	A vs. O: OR = 1.18, 95% CI = 1.03–1.36, *p* = 0.019
	713 EOC patients (USA) (71)	Blood type A/A2 allele	Prognosis/Overall survival (OS)	↑	A vs. Non-A: HR = 0.77, 95% CI = 0.63-0.94; A2 allele vs. O allele: HR = 0.50, 95% CI = 0.25–0.99
Bladder cancer	931 primary NMIBC patients (Multi-center) (74)	Blood type A, blood type B	Prognosis/Risk of recurrence, progression	↓	A vs. O: SHR = 0.77, 95% CI = 0.62-0.95, *p* = 0.015; SHR = 0.57, 95% CI = 0.34–0.95, *p* = 0.031 B vs. O blood type: SHR = 0.60, 95% CI = 0.42–0.85, *p* = 0.004; SHR = 0.42, 95% CI = 0.16–1.09, *p* = 0.075
	2,086 MIBC patients treated with cystectomy (USA) (75)	Non-O blood type	Prognosis/5-year recurrence-free survival	↓	Non-O vs. O: RFS: 65% vs. 69%, *p* = 0.04; CSS: 64% vs. 70%, *p* = 0.02
		Blood type A	Mortality rate	↑	A vs. Non-A: HR = 1.22, 95% CI = 1.04-1.44, *p* = 0.01
	74 Bladder cancer patients vs. 142 healthy donors (Croatia) (76)	O1 and A1 alleles	Grade histology;recurrence rates	↑	O1:71.4%, 70.2%; *p* = 0.019 A1:70.1%, 74.2%; *p* = 0.007
		A2 allele	Metastasis ratio	↓	0%, *p* = 0.024
		O1 and O2 alleles	Infiltration	↑	55.1%, *p* < 0.001; 100%, *p* = 0.045

“^*^”: ↑ indicates that the corresponding risk of the blood group population is relatively increased, and ↓ indicates that the risk is relatively decreased.

### 3.2 Cardiovascular diseases

#### 3.2.1 Atherosclerotic diseases

Accumulating evidence points to a discernible association between the ABO blood group and both coronary artery calcification (CAC) and plaque vulnerability. In a CAC study, Wang et al. enrolled 301 patients undergoing coronary CT angiography, categorizing them into non-calcification (*n* = 197) and calcification (*n* = 104) groups according to the coronary artery calcium score (CACS). Univariate logistic regression analysis demonstrated a significantly higher proportion of blood type A in the calcification group (35.58%) vs. the non-calcification group (21.32%, *p* = 0.008), while type O was considerably less frequent (20.19% vs. 31.47%, *p* = 0.037). Multivariate logistic regression further established blood type A as an independent risk factor for CAC (OR = 2.217, *p* = 0.006), implying a potential role in the early pathogenesis of atherosclerosis (77).

Extending the analysis to plaque vulnerability of acute coronary syndrome (ACS), optical coherence tomography (OCT) studies have revealed that patients with non-O blood types are more prone to exhibit high-risk plaque features. Specifically, these patients displayed a larger lipid arc (288.57° ± 63.51° vs. 263.69° ± 59.40°, *p* = 0.004), a thinner fibrous cap (68.7 ± 14.2 μm vs. 84.0 ± 16.4 μm, *p* < 0.001), and a greater prevalence of thin-cap fibroatheroma (TCFA) (24.3% vs. 5.0%, *p* < 0.001). This association was most evident in patients with blood type A, who presented with the most pronounced features—notably the thinnest fibrous caps (64.7 ± 14.3 μm) and the highest frequency of TCFA (30.5%; *p* < 0.001) (78). The presented evidence is consistent with a role for ABO blood groups in atherosclerosis, indicating that non-O types (especially type A) have been associated with an increased likelihood of both coronary artery calcification and vulnerable plaque phenotypes.

#### 3.2.2 Thrombotic diseases

The protective effect of blood group O against venous thromboembolism (VTE) was noted as early as 1971, with its prevalence disproportionately low in cases of pregnancy and postpartum pulmonary embolism or deep vein thrombosis; carriers had about half the relative risk of non-O individuals (79). A large-scale investigation, involving 1.5 million Swedish and Danish blood donors, further demonstrated a significantly elevated overall VTE risk among non-O blood groups (IRR = 1.80). This risk was most pronounced for pregnancy-associated VTE (IRR = 2.22), followed by deep vein thrombosis (IRR = 1.92) and pulmonary embolism (IRR = 1.75). The population attributable risk percentage (PAR%) for VTE due to non-O blood groups reached 32% (80). Subsequent investigations have further delineated this association at the genotypic level. Research by Goumidi et al. on a cohort of 5,425 VTE patients and 8,445 controls identified a clear gradation of VTE risk among ABO haplotypes. The A1 and B haplotypes were associated with substantially elevated risks (OR = 1.78 and 1.76, respectively), whereas the A2 haplotype was linked to a more modest increase (OR = 1.16). In contrast, the O2 haplotype demonstrated a significant protective association (OR = 0.70) (81). Analyses of the UK Biobank data corroborated a dose-dependent effect of the A allele, demonstrating that each additional allele incrementally elevates the hazard for VTE (HR = 1.273) (82). Extending these observations to other prothrombotic settings, a separate study investigated the role of ABO blood type in persistently antiphospholipid antibody (aPL)-positive patients. In a retrospective cohort of 226 persistently antiphospholipid antibody (aPL)-positive patients, Shusterman et al. examined the association between ABO blood type and venous thromboembolism (VTE). Overall, non-O blood type was not significantly associated with VTE (OR 1.64, 95% CI 0.94–2.88, *P* = 0.08), but a significant interaction with sex was observed (*P* = 0.057). Stratified analysis revealed that non-O blood type was associated with a markedly increased VTE risk in men (OR 4.94, 95% CI 1.37–17.85, *P* = 0.02), whereas no such association was found in women (OR 0.96, 95% CI 0.50–1.83, *P* = 0.52). These findings suggest that non-O blood type may represent an underrecognized risk factor for VTE specifically among aPL-positive men (83). Further supporting this link in a more homogeneous patient population, a subsequent study focused on primary antiphospholipid syndrome (PAPS). In 70 patients with primary antiphospholipid syndrome (PAPS), non-O blood type was associated with a higher frequency of venous thrombosis compared to O type (72.7% vs. 46.2%, *p* = 0.040). vWF antigen and activity levels were significantly lower in the O group (vWF: Ag 75.54 ± 8.68 vs. 79.51 ± 7.07 IU/dL, p=0.041; vWF: CB 70.23 ± 11.96% vs. 77.92 ± 13.67%, *p* = 0.020), with no differences in antiphospholipid antibody profiles or traditional risk factors. These findings indicate that ABO blood group influences thrombotic phenotype in PAPS, with non-O favoring venous events (84). The relevance of ABO blood groups extends beyond general VTE risk to specific thrombotic disorders. For instance, non-O blood groups may increase the risk of vascular involvement in Behçet's disease (BD). In a retrospective analysis of 411 BD patients, non-O blood groups (A, B, AB) accounted for 65.2% of the cohort and were linked to a significantly higher frequency of venous involvement compared with blood group O (35.4% vs. 24.5%; *p* = 0.023). After multivariable adjustment, non-O groups showed an approximately two-fold increased risk for overall vascular and venous involvement, as well as for arterial disease. Among non-O types, blood group B carried the highest risk. These findings indicate that non-O blood groups may represent independent risk factors for vascular BD, potentially contributing to the prothrombotic tendency observed in these patients (85). Taken together, non-O blood groups—particularly the A1 and B haplotypes—have been associated with an increased susceptibility to VTE. This association appears most prominent in pregnancy-related VTE, and a dose-dependent relationship with the A allele has been suggested.

#### 3.2.3 Ischemic heart disease

Emerging epidemiological evidence raises the possibility of an association between ABO blood groups and the risk of ischemic heart disease (IHD). Earlier studies have indicated a higher occurrence of blood types A and B in IHD patients, along with a lower prevalence of type O. Non-O blood groups appear to confer a moderately increased relative risk for IHD, ranging from 1.31 to 1.34, which may rise to approximately 1.47 among patients with myocardial infarction (MI) (86). Consistent patterns have been observed in studies of early-onset ischemic stroke (EOS). A GWAS meta-analysis (*n* = 16,730) tied the O1 subtype to lower EOS risk (OR = 0.88) and the A1 subtype to higher risk (OR = 1.16) (87). Similarly, non-O types (especially B) raise susceptibility to ischemic stroke and peripheral artery disease, while the O1 allele may be protective (adjusted OR = 0.27) (88). Regarding MI, the BB genotype has been robustly associated with risk. In a cross-sectional study involving 550 participants (275 acute MI patients and 275 healthy controls), the BB genotype was more prevalent among MI patients (13.8%) than among controls (6.2%, *p* = 0.004). After multivariate adjustment, individuals with the BB genotype exhibited a 3.32-fold higher odds of MI compared with those carrying the OO genotype (AOR = 3.32, 95% CI = 1.36–8.08) (89). Another study reported that both diplotypes (OR = 1.23, 95% CI = 1.05–1.44) and haplotypes (OR = 1.18, 95% CI = 1.04–1.35) containing the A1 allele were associated with an elevated risk of MI (90). Therefore, non-O blood types, including the BB genotype and the A1 allele, have been reported to be associated with increased susceptibility to myocardial infarction and ischemic heart disease.

#### 3.2.4 Congenital heart disease

A six-year cohort study encompassing 39,042 participants identified a significant inverse association between blood type A and the risk of isolated congenital heart disease (CHD) (OR = 0.82, 95% CI = 0.78–0.87). This protective association was consistently observed across principal CHD subtypes, including ventricular and atrial septal defects (91). These results implicate the ABO blood group as a potential biomarker for isolated CHD susceptibility, thereby suggesting its role in disease etiology; however, the precise biological mechanisms demand further elucidation (Table 2).

**Table 2:** Summary of studies evaluating the relationship between ABO blood group and cardiovascular diseases.

Disease category	Study population	Associated blood type(s)/gene(s)	Aspect involved	Level of association	Effect estimate (95% CI)
Coronary Artery Calcification (CAC)	301 suspected CHD patients (China) (77)	Blood type A	Percentage of calcium	↑	A vs. Non-A: OR = 2.217, 95% CI: 1.260–3.900, *p* = 0.006
Acute Coronary Syndrome (ACS)	257 ACS patients (China) (78)	Non-O blood type	Lipid arc	↑	Non-O vs. O: 88.57°± 63.51° vs. 263.69°± 59.40°, *p* = 0.004
			Fibrous cap	↓	Non-O vs. O: 68.7 ± 14.2 μm vs. 84.0 ± 16.4 μm, *p* < 0.001
			Thin-cap fibroatheroma (TCFA)	↑	Non-O vs. O: 24.3% vs. 5.0%, *p* < 0.001
Venous Thromboembolism (VTE)	1,112,072 blood donors (Sweden/Denmark) (80)	Non-O blood type	Susceptible	↑	Non-O vs. O: IRR = 1.80, 95% CI: 1.71–1.88, *p* < 0.001
	5,425 VT patients vs. 8,445 controls (European ancestry) (81)	A1,B allele	Susceptible	↑	A1 vs. O1 allele: OR = 1.78, 95% CI: 1.67–1.92, *p* = 3.15 × 10^−60^; B vs. O1 allele: OR = 1.76, 95% CI: 1.58–1.96, *p* = 1.88 × 10^−24^
	326,526 UK Biobank participants (O and A blood groups) (82)	A allele	Susceptible	↑	For each additional A allele: HR = 1.273, 95% CI: 1.245–1.302, *p*[FDR] = 4.43 × 10^−96^
	70 PAPS patients (Brazil) (84)	Non-O blood type	Susceptible	↑	Non-O vs. O: 72.7% vs. 46.2%, *p* = 0.040
	411 BD patients (Turkey) (85)	Non-O blood type	Susceptible	↑	Non-O vs. O: 35.4% vs. 24.5%, *p* = 0.023
Ischemic Heart Disease (IHD)	792 IHD patients vs. 6,366 controls (South Africa) (86)	Non-O blood type	Susceptible	↑	Non-O vs. O: RR = 1.31~1.34 (*p* < 0.001)
Early-Onset Ischemic Stroke (EOS)	16,730 EOS cases vs. 599,237 controls (Multi-national) (87)	A1 allele	Susceptible	↑	A1 allele vs. O1 allele: OR = 1.16, 95% CI: 1.11–1.21, *p* = 6.54 × 10^−13^
Ischemic Stroke and Peripheral Artery Disease	81 IS/PAD patients vs. 201 controls (Brazil) (88)	Blood type B	Susceptible	↑	B vs. Non-B: OR = 2.94, 95% CI: 1.38–6.24, *p* < 0.01
Myocardial Infarction (MI)	275 AMI patients vs. 275 controls (Pakistan) (89)	BB genotype	Susceptible	↑	BB vs. OO: AOR = 3.32, 95% CI: 1.36–8.08, *p* = 0.008
Isolated Congenital Heart Disease (CHD)	19,795 CHD cases vs. 19,247 controls (China) (91)	Blood type A	Susceptible	↓	A vs. O: OR = 0.82, 95% CI: 0.78–0.87, *p* < 0.001

### 3.3 Infectious diseases

#### 3.3.1 Helicobacter pylori

In a comprehensive meta-analysis encompassing 30 studies and 12,708 participants, blood type O was identified as a risk factor for *Helicobacter pylori (H. pylori)* infection, exhibiting a significant 16.3% increase in odds compared to non-O blood groups (pooled OR = 1.163, 95% CI = 1.074–1.259, *p* < 0.001). Conversely, blood types B and AB demonstrated protective effects, with respective risk reductions of 17% and 29% (B: OR = 0.831, *P* = 0.002; AB: OR = 0.709, *p* < 0.001) (92), thereby identifying the ABO system as a significant modifier of infection susceptibility. This pattern of blood group-dependent susceptibility is corroborated at the molecular level. An investigation involving 290 Pakistani patients identified the highest *H. pylori* infection rate (43/90; 47.8%) in non-secretor individuals with blood type O. This subgroup also harbored a disproportionately high prevalence (72%) of BabA-positive *H. pylori* strains. Clinically, non-secretor status was positively correlated with symptom severity, with 62% of patients presenting composite symptoms being non-secretors (93). These findings collectively indicate that individuals with blood type O, particularly non-secretors, harbor a substantially increased risk of *H. pylori* infection.

#### 3.3.2 Noroviruses

Noroviruses (NoVs) are a major cause of acute gastroenteritis in both adults and children. Host susceptibility to NoV infection has been postulated to correlate with ABO blood group status (94, 95). A meta-analysis encompassing 17 studies (2,304 participants) determined that individuals with blood type O exhibit a 28% significantly elevated risk of Norovirus infection (pooled OR = 1.28, 95% CI = 1.03–1.59, *p* = 0.03). In contrast, no statistically significant association with infection risk was observed for blood types A (OR = 0.90, *p* = 0.37), B (OR = 0.85, *p* = 0.25), or AB (OR = 0.91, *p* = 0.67) (96).

Molecular epidemiological research further elucidates the influence of ABH antigen secretor status on this blood group-dependent susceptibility. A 1:1 matched analysis of 147 Taiwanese children with Norovirus infection and their healthy controls revealed that individuals carrying the weak-secretor FUT2 gene mutation (A385T) were significantly underrepresented in the case group (5.4% vs. 23.1%). Conversely, those with the wild-type FUT2 genotype (secretors) faced a markedly higher infection risk, being 6.766 times more likely to contract the virus (matched OR = 6.766, *p* < 0.0001) (97). These findings suggest that norovirus infection risk is influenced by both ABO blood type and antigen secretor status. Although blood type O has been associated with increased susceptibility, this risk is further amplified in individuals who express the ABH antigen secretor phenotype.

#### 3.3.3 Cholera

A consistent association between ABO blood group and susceptibility to cholera has been observed across diverse Asian populations. An investigation in the Philippines demonstrated a marked disparity in ABO blood group distribution between cholera cases and controls. Blood type O was significantly overrepresented in the case group (64.4% vs. 44.6%-46.4%), whereas type A was substantially underrepresented (10.3% vs. 25.8%-27.9%) (98). Concurrently, an analysis from Kolkata, India, confirmed a significantly elevated frequency of type O among patients infected with Vibrio cholerae compared to controls (44.3% vs.32.4%; χ^2^=8.31, *p* < 0.05). Notably, this blood group association was specific to cholera infection and was not observed in *Vibrio parahaemolyticus* cases (99). Further evidence from a large-scale study in Bangladesh demonstrated that individuals with blood type O not only exhibited a greater risk of *V. cholerae* infection (57% of patients vs. 30% of controls, *p* < 0.01) but also developed more severe disease manifestations (68% of severe diarrhea cases vs. 36% of asymptomatic cases, *p* < 0.01). In contrast, blood type AB appeared to confer a protective effect, being rare among patients (1% vs. 9% in controls) (100). This association extends to O139 serogroup cholera, with type O comprising 64% of patients (vs. 34% in controls, *p* < 0.0001) and no AB individuals identified among cases (vs. 8% in controls) (101). The collective evidence from these studies suggests that blood type O is a major risk factor for cholera (Table 3).

**Table 3:** Summary of studies evaluating the relationship between ABO blood group and infectious diseases.

Disease category	Study population	Associated blood type(s)/gene(s)	Aspect involved	Level of association	Effect estimate (95% CI)
Helicobacter pylori	12,708 subjects (Global/Meta-analysis) (92)	Blood type O	Susceptible	↑	O vs. Non-O: OR = 1.163, 95% CI = 1.074–1.259, *p* < 0.001
	290 symptomatic patients (Pakistan) (93)	Blood type O (Non-secretory type)	Susceptible	↑	47.8%(43/90)
Noroviruses	2,304 participants (Global/Meta-analysis) (96)	Blood type O	Susceptible	↑	O vs. Non-O: OR = 1.28, 95% CI = 1.03–1.59, *p* = 0.03
	147 infected children vs. 147 controls (Taiwan, China) (97)	Secretory type	Susceptible	↑	Secretory type vs. Non-secretory type: OR = 6.766, 95% CI = 2.649–17.285, *p* < 0.0001
Cholera	87 cholera cases (Philippines) (98)	Blood type O	Susceptible	↑	Proportion of patients with blood group O vs. proportion of healthy controls with blood group O: 64.4% vs. 44.6%−46.4%
	402 acute gastroenteritis patients (India) (99)	Blood type O	Susceptible	↑	Proportion of patients with blood group O vs. proportion of healthy controls with blood group O: 44.3% vs. 32.4%, χ^2^ = 8.31, *p* < 0.05
	10,000 subjects (Bangladesh) (100)	Blood type O	Susceptible	↑	Proportion of patients with blood group O vs. t proportion of healthy controls with blood group O: 57% vs. 30%, *p* < 0.01
			Poor prognosis	↑	Proportion of blood type O in critically ill patients vs. proportion of blood group O in asymptomatic patients: 68% vs. 36%, *p* < 0.01
	1,298 subjects (Bangladesh) (101)	Blood type O	Susceptible	↑	64% vs. 34%, *p* < 0.0001

## 4 ABO blood group and therapeutic response: efficacy variation and risk stratification

### 4.1 Cancer therapeutics

Current cancer therapeutics face considerable challenges, particularly in accurately predicting treatment response (102, 103). In this context, the ABO blood group system has attracted growing attention as a potential biomarker for therapeutic efficacy prediction. Emerging evidence indicates that ABO phenotype may modulate the response to both chemotherapy and immunotherapy across different cancer types.

A multicenter cohort study conducted in Japan enrolled 1,153 patients who underwent curative resection for pancreatic cancer. A significant interaction was observed between ABO blood group and adjuvant chemotherapy regimen (no chemotherapy, S-1-based, or gemcitabine-based) for both disease-free survival (DFS; *P*_interaction_ = 0.011) and pancreatic cancer-specific survival (PCSS; *P*_interaction_ = 0.008). Among patients who did not receive adjuvant therapy, those with blood types B and AB had a higher recurrence risk (DFS HR = 1.65 and 1.79, respectively; both *P* < 0.05). In contrast, within the S-1–treated subgroup, blood type AB was associated with a reduced recurrence risk (HR = 0.63, 95% CI 0.39–1.00), suggesting a potential predictive value of ABO typing for S-1 chemotherapy response in pancreatic cancer (104). Consistently, in a study of 82 patients with PD-L1 high–expressing (TPS ≥50%) metastatic non-small cell lung cancer (NSCLC) treated with pembrolizumab monotherapy, blood type O emerged as an independent favorable prognostic factor. Compared with non-O types, patients with blood type O exhibited significantly prolonged overall survival (HR = 0.22, *P* = 0.037; median 62 vs. 19 months) and progression-free survival (HR = 0.21, P = 0.024; median 39 vs. 4 months). Notably, no such association was observed in patients receiving combination immunotherapy or chemotherapy (28). Current evidence suggests that the ABO blood group plays a dual role in cancer, affecting both disease risk and treatment efficacy.

### 4.2 Circulatory disease therapeutics

Anticoagulation management is confronted by substantial interindividual variability in therapeutic response, necessitating complex dosage individualization. The ABO blood group system has been reported to serve as a genetic modifier contributing to this variability. A clinical cohort study demonstrated that warfarin-treated patients with deep vein thrombosis or atrial fibrillation who had blood type O required significantly lower maintenance doses to attain target INR compared to their non-O counterparts (mean 2.74 ± 0.83 mg vs. 3.19–3.32 mg; *p* < 0.05). The physiological basis for this observation lies in the inherently lower plasma concentrations of coagulation factor VIII and von Willebrand factor in type O individuals, which potentiates warfarin's effect. These results provide a compelling rationale for integrating ABO blood group status into algorithms for warfarin dose optimization (25).

The ABO blood group system has been reported to affect therapeutic efficacy in both antiplatelet and vasoactive treatments. A prospective cohort study of 3,039 patients with comorbid coronary artery disease and type 2 diabetes identified blood type O as an independent risk factor for clopidogrel-related low platelet reactivity (LTPR) (adjusted OR = 1.298). Conversely, type A was identified as a protective factor (adjusted OR = 0.804) (26). In a multicenter cohort study of vasoactive therapies involving 83 neonates with persistent pulmonary hypertension of the newborn (PPHN) treated with inhaled nitric oxide (iNO) monotherapy, those with blood type O exhibited the most favorable oxygenation response, achieving a treatment response rate of 87.2%. These infants demonstrated the lowest oxygenation index (OI = 3.9, *p* = 0.012) and the highest PaO_2_/FiO_2_ ratio (325.0, *p* = 0.009). In contrast, neonates with blood type A showed the poorest treatment response (105).

The ABO blood group emerges as a key modulator of drug response across different classes of medication. While type O heightens the risk of low platelet reactivity with clopidogrel, it paradoxically predicts superior responsiveness to nitric oxide therapy in neonatal pulmonary hypertension. This contrast highlights its dual role and suggests that ABO typing may serve as a potential biomarker for guiding personalized pharmacotherapy.

### 4.3 Infectious disease therapeutics

Antituberculosis drug-induced liver injury (ATLI) constitutes a significant adverse effect of tuberculosis management, potentially causing treatment discontinuation, extended duration, and heightened risk of drug resistance. Recent research proposes the ABO blood group system as a potential modulator of ATLI risk in patients receiving rifampin-based regimens. Clinical analyses revealed that, relative to blood type O, types A (OR = 2.014, HR = 1.815) and B (OR = 2.023, HR = 1.845) were associated with a significantly increased risk. The most pronounced susceptibility was observed in type AB individuals, conferring the most substantial risk elevation (OR = 2.696, HR = 2.557) (27). These collective results imply that non-O blood groups, with type AB being paramount, are linked to an elevated risk of developing ATLI during rifampin therapy.

### 4.4 Digestive disease therapeutics

The management of Crohn's disease with infliximab presents considerable clinical challenges, including primary non-response, secondary loss of response, and significant pharmacokinetic variability. The absence of predictive biomarkers further impedes treatment individualization. Growing evidence establishes the ABO blood group as a significant modifier of Infliximab (IFX) therapeutic outcomes. A cohort analysis of 293 Crohn's disease patients, including 107 on IFX with a minimum 2-year follow-up, demonstrated no association between ABO status and disease development risk. However, a significant association with therapeutic efficacy was established. Relative to type O individuals, those with blood type AB exhibited a pronounced treatment benefit, characterized by significantly higher mucosal healing rates at both 1-year (OR = 4.42, *p* = 0.044) and 2-year (OR = 5.20, *p* = 0.039). The response of type B patients did not differ significantly from the type O reference group (106).

Mounting evidence positions ABO typing as a promising predictive biomarker for IFX outcomes in CD. This paves the way for blood group-stratified treatment, which would optimize precision medicine and resource use. A simple yet effective strategy would prioritize IFX for type AB patients and mandate closer surveillance for type A patients to mitigate the risk of treatment failure (Table 4).

**Table 4:** Association of ABO blood groups with response to drug therapy in related diseases.

Therapeutic area	Drug/class	Study population	Observed effect/outcome	Associated blood types	Level of association	Effect estimate (95% CI)
Pancreatic cancer	S-1 chemotherapy	1,153 resected PC patients (Japan) (104)	Disease-free survival (DFS)	Blood type AB	↓	AB vs. Non-AB: HR = 0.63, 95% CI 0.39–1.00, *p* = 0.05
Non-small cell lung cancer (NSCLC)	Pembrolizumab Monotherapy	82 NSCLC patients (ICI monotherapy) vs. 36 controls (Germany) (28)	Overall survival (OS), progression-free survival (PFS)	Blood type O	↑	vs. Non-O (OS): HR = 0.22, 95% CI: 0.1–0.9, *p* = 0.037; O vs. Non-O (PFS): HR = 0.21, 95% CI: 0.1–0.8, *p* = 0.024
Deep vein thrombosis and atrial fibrillation	Warfarin	358 DVT/AF patients on warfarin (China) (25)	Dose of warfarin	Blood type O	↓	O: 2.74 ± 0.83 mg/day; A: 3.19 ± 0.97 mg/d (vs. O, *p* < 0.05); B: 3.32 ± 0.97 mg/d (vs. O, *p* < 0.01); AB: 3.14 ± 0.93 mg/d (vs. O, *p* < 0.05);
Coronary artery disease with type 2 diabetes mellitus	Clopidogrel	3,039 CAD/T2DM patients on clopidogrel (China) (26)	Low platelet reactivity (LTPR)	Blood type O	↑	O vs. Non-O: OR = 1.298,95% CI: 1.099–1.534, *p* = 0.002
Persistent pulmonary hypertension of the newborn (PPHN)	Inhaled Nitric Oxide (iNO)	83 PPHN neonates treated with iNO (China) (105)	Treatment response rate	Blood type O	↑	O: 34 cases (87.2%) A: 19 cases (79.2%) B: 10 cases (50.0%) (the lowest response rate)
Tuberculosis	Rifampin	146 ATLI cases vs. 584 controls (China) (27)	Antituberculosis drug-induced liver injury (ATLI)	Blood type AB	↑	A: OR = 1.832, 95% CI: 1.126–2.983, *p* = 0.015; B: OR = 1.751, 95% CI: 1.044–2.937, *p* = 0.034; AB: OR = 2.059, 95% CI: 1.077–3.938, *p* = 0.029; Non-O (A+B+AB): OR = 1.822 95% CI: 1.173–2.831, *p* = 0.007
Crohn's disease	Infliximab (IFX)	293 CD patients treated with IFX (China) (106)	Mucosal healing rates	Blood type AB	↑	1-year: OR = 4.42,95% CI:1.04–18.76, *p* = 0.044; 2-year: OR = 5.20,95% CI: 1.09–24.86, *p* = 0.039

## 5 Discussion

The persistence of the ABO blood group polymorphism throughout human evolution, without any single type being categorically selected against, is a compelling example of balanced selection (107). This evolutionary model posits that the specific advantages and disadvantages associated with each blood type are context-dependent, creating a dynamic equilibrium that maintains diversity within the population (108). For instance, while non-O blood groups are well-documented to confer an elevated risk for certain pathologies, such as malignancies and thromboembolic diseases, they simultaneously appear to offer a protective advantage against severe manifestations of specific infections, notably those caused by Helicobacter pylori and norovirus (57, 109, 110). Conversely, the O blood group, despite its association with increased susceptibility to these and other gastrointestinal infections, demonstrates a relative resistance to the development of certain cardiovascular and neoplastic conditions (111, 112). This trade-off—where the genetic determinant that predisposes to one class of diseases may grant resilience against another—likely underpins the evolutionary success and sustained prevalence of the ABO system. Regarding population and ethnic differences, the associations between ABO blood groups and disease susceptibility exhibit both consistency and significant variation across different human populations. This is primarily attributable to the combined effects of inherent differences in allele frequencies and environmental selective pressures. For instance, the association between non-O blood types and pancreatic cancer risk appears to be universal across populations, whereas the ethnic differences observed in susceptibility to VTE and cholera suggest the influence of local selective pressures (113–115). Rather than being “good” or “bad,” each blood type represents a different evolutionary strategy for survival, with its net fitness contingent upon the prevailing environmental and pathogenic pressures.

The functional paradigm of the ABO blood group system has expanded far beyond its initial role in transfusion safety. ABO antigens are now recognized as intrinsic regulators of pathophysiology, modulating disease risk, clinical prognosis, and therapeutic efficacy (116–118). This unifying role establishes the ABO system as a fundamental biological framework that connects disease etiology with clinical intervention, offering a dual utility for risk stratification and therapy personalization. However, the field remains characterized more by phenomenological observations than mechanistic insights. While large-scale epidemiological studies have robustly established correlations, they often fall short of explaining the underlying “why” (119). Critical questions still persist, why are non-O groups linked to certain cancers and cardiovascular diseases, while the O group is tied to gastrointestinal infections. Furthermore, research often siloes “blood group-disease” and “blood group-drug response” associations, overlooking potential common pathways. For example, could the same mechanism that increases pancreatic cancer risk in non-O individuals also affect their response to chemotherapy.

Compared to the long-standing study of disease links, exploring ABO in drug response is a more nascent field. Consequently, elucidating the shared mechanisms through which ABO influences both disease development and drug responses is of paramount importance. This integrated perspective not only holds great theoretical value but also provides an actionable pathway for advancing precision medicine.

**Figure 4:**
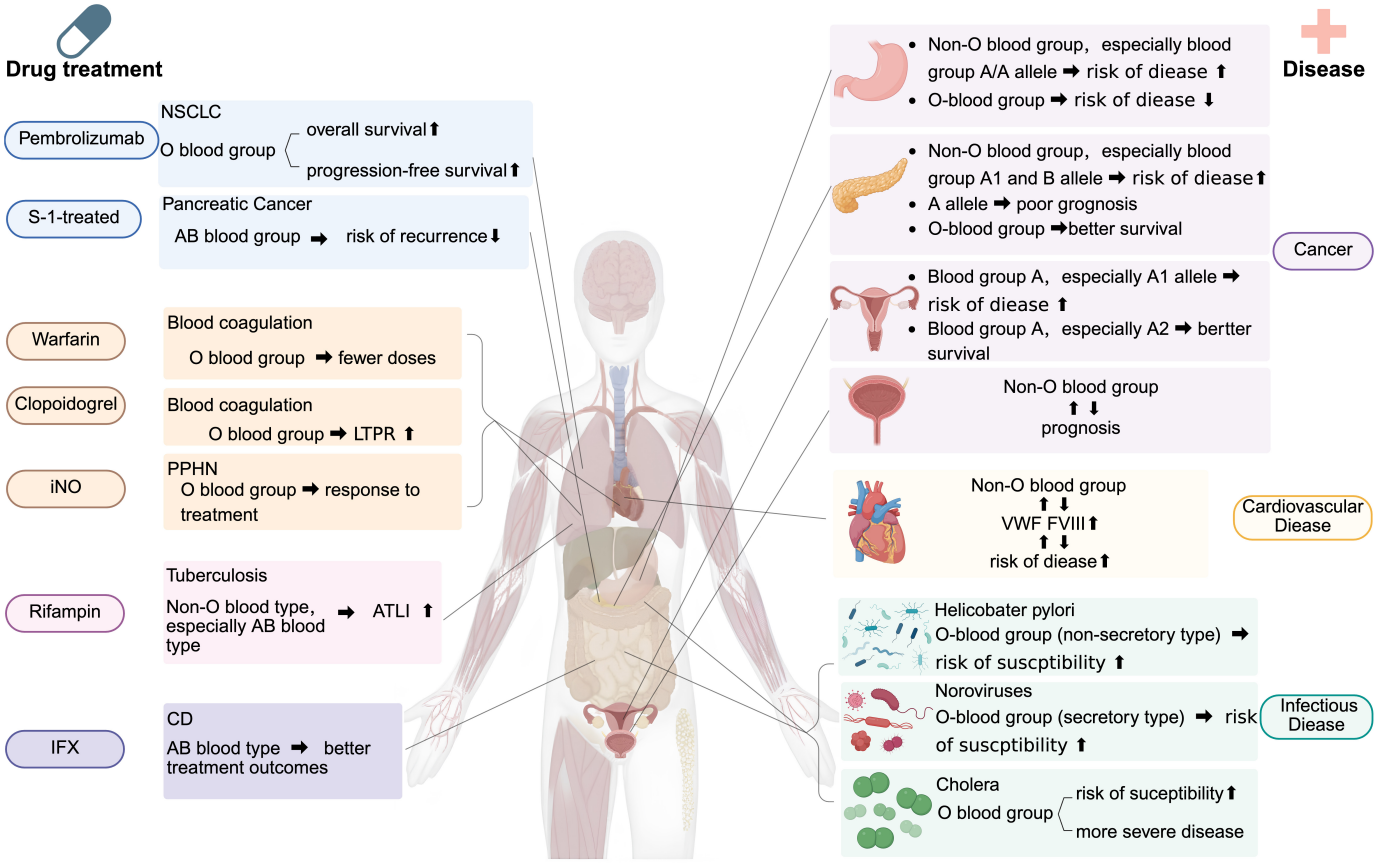
Systemic associations of ABO blood groups with diseases and therapeutic responses. This schematic illustrates how ABO blood groups influence organ-specific disease susceptibility, clinical prognosis, and response to pharmacological interventions at a systemic level.

This review systematically synthesizes current knowledge on the roles of ABO blood groups in disease risk and therapeutic responses, aiming to move beyond mere statistical associations to illuminate their substantial impact on pathogenesis and drug efficacy. Building on this synthesis, we propose a paradigm shift to reevaluate ABO blood groups through an integrated disease-therapy lens. In this view, ABO is not merely a static genetic marker of susceptibility but also a dynamic functional element involved in pathophysiological regulation, mechanistically linking disease vulnerability and pharmacological effects at the molecular level. Potential applications range from early screening and risk stratification for high-risk populations, to guiding blood group-informed medication choices, and facilitating prognosis assessment—ultimately enabling blood group-stratified patient management for truly precision healthcare (Figure 4).

## 6 Conclusion

The ABO blood group system is determined by specific glycosyltransferases that catalyze the addition of distinct terminal sugars to the H antigen, thereby generating characteristic blood group antigens. The ABO blood types are closely associated with disease susceptibility, progression, and drug response. The ABO blood groups exhibit pleiotropic associations with disease risk: non-O types demonstrate an increased propensity for specific malignancies of gastric and pancreatic cancers and cardiovascular disorders, whereas the O phenotype is linked to a heightened vulnerability to particular gastrointestinal infections caused by Helicobacter pylori and norovirus. Concurrently, therapeutic responses across a spectrum of pathologies show significant variation by ABO status. Despite these observations, the field remains characterized by fragmented correlative studies and a pronounced lack of integrated mechanistic investigation. This review synthesizes extant evidence to propose a paradigm shift toward a unified understanding of ABO pathophysiology, bridging disease etiology and therapeutic response. A critical future direction involves delineating the molecular mechanisms and exploring the translational potential of the ABO system to advance precision medicine.
